# Brain Blindness Versus Eye Blindness: Genetic and Molecular Connections Between Alzheimer’s Disease and Visual Impairment

**DOI:** 10.3390/cimb48101081

**Published:** 2026-10-19

**Authors:** Eva Bagyinszky, Seong Soo A. An

**Affiliations:** 1 College of BioNano Technology, Gachon University, Seongnam 13120, Republic of Korea; eva85@gachon.ac.kr; 2 Department of Bionano Technology, Gachon Medical Research Institute, Gachon University, Seongnam 13120, Republic of Korea

**Keywords:** Alzheimer’s disease, visual impairment, genetics, mutations, posterior cortical atrophy (PCA), glaucoma, age-related macular degeneration

## Abstract

Visual dysfunctions were observed among Alzheimer’s disease (AD) patients, including posterior cortical degeneration, retinal abnormalities, or coexisting ocular disease. Posterior cortical atrophy (PCA) is a clinical syndrome characterized by progressive higher-order visual impairment and is most frequently associated with AD pathology, although other neurodegenerative causes may be possible. Pathogenic variants in PSEN1 and PSEN2 were reported in individual cases of PCA and may support a possible monogenic contribution to selected early-onset posterior phenotypes. By contrast, variants and common AD risk genes, including GRN, MAPT, TREM2, BIN1, PICALM, ABCA7, and APOE E4 allele, represented broader genetic associations whose specific contribution to posterior cortical vulnerability remains incompletely defined. These forms of evidence should be interpreted separately. Genome-wide association studies identified overlapping loci associated with AD and glaucoma, including loci on chromosome 11 involving MTCH2, NDUFS3, and SPI1. This overlap reflected shared genetic susceptibility, pleiotropy, or putative convergent biological pathways involving mitochondrial function, immune regulation, and cellular stress. However, shared loci and pathway associations did not establish direct signaling, anatomical communication, or a causal relationship between brain and ocular tissues. The opposing tissue-specific effects reported for APOE further indicated that data in the brain may not be extrapolated to the retina. Future studies should clarify the cell-specific effects, causal direction, and clinical significance of shared genetic factors using well-characterized cohorts and tissue-specific experimental models. Overall, current evidence supported the partially overlapping genetic and cellular mechanisms in AD and selected ocular disorders, while further studies should be needed on the biological relationship between the brain and eye.

## 1. Introduction: Epidemiological and Pathological Links Between AD and Visual Impairment

While the main hallmark of Alzheimer’s disease (AD) is memory loss, visual impairment was also commonly reported in AD patients [1,2]. These impairments could range from basic sensory deficits to complex processing issues, and patients with AD experienced critical vision loss even without major eye health issues, due to the damage to the visual processing center in the brain [3,4].

Posterior cortical atrophy (PCA) was a clinical-radiological neurodegenerative syndrome. Posterior cortical atrophy (PCA) was a clinical-radiological neurodegenerative syndrome characterized by progressive impairment of higher-order visual functions, particularly visuospatial and visuoperceptual abilities. Memory and language may be relatively preserved in the early stages, although they could be affected during the disease progression. AD was found as the underlying pathology in the vast majority of PCA cases, which could be referred to as PCA due to AD (PCA-AD). Neuroimaging commonly showed involvement of posterior cortical regions, including the parietal, posterior temporal, and occipital cortices. Visual hallucinations were also common in patients with PCA [5].

PCA should therefore be distinguished from its underlying pathology. PCA due to AD pathology (PCA-AD) referred to PCA in which AD pathology is established or considered the primary underlying cause. Non-AD PCA or PCA-like phenotypes referred to patients with a PCA-type clinical presentation in whom another neurodegenerative pathology was identified or the underlying pathology remained uncertain. The consensus framework separated the syndrome-level diagnosis of PCA from the disease-level classification of its underlying pathology. Cortical blindness was also distinct from PCA because it denoted severe visual loss caused by dysfunction of the posterior visual cortex or visual pathways, rather than a specific progressive neurodegenerative syndrome [1,2,3,4,5,6,7].

PCA should be distinguished from its underlying pathology. PCA-AD referred to PCA in which AD pathology was established or considered the primary cause. Non-AD PCA or PCA-like phenotypes referred to patients with a PCA-type clinical presentation in whom another neurodegenerative pathology was identified or the underlying pathology remained uncertain. Visual hallucinations were described in PCA-AD, but no significant differences were detected between PCA-AD and non-AD PCA [1,2,3,4,6].

An epidemiological link was identified between glaucoma and AD. Studies suggested that patients with glaucoma could have an increased risk for developing AD or vascular dementia, especially in older ages [8]. Furthermore, glaucoma was more prevalent in AD patients, compared to the general population. Studies reported that approximately 24.5% to 25.9% of AD patients presented glaucoma, while the frequency of glaucoma in control groups was less than 10% [9,10,11,12]. Glaucoma and AD were suggested to share pathological hallmarks, since amyloid beta (Aβ) and tau aggregates were detected in the brain and retina, respectively [13]. Cell studies of RGC-5 photoreceptors revealed that Aβ could be toxic to retinal cells, since treatment of retinal ganglion cell line with Aβ25-35 fragment promoted cell death. Also, the treatment with 25–35 increased the levels of amyloid precursor protein (APP) and Aβ42 in both RGC-5 and mouse cortical neuron cultures [14]. A pathological link was found between AD and cataracts, since Aβ could be accumulated in the lenses of patients with AD and could form equatorial supranuclear cataracts [15]. The presence of Aβ (Aβ40 and Aβ42) peptides was confirmed to be present in the cytosol of lens fiber cells at concentrations comparable to those reported in the brain. These Aβ peptides could interact with cytosolic lens proteins, including alphaB-crystallin, and promote the specific protein aggregation and lens opacification [15,16,17].

AD and age-related macular degeneration (AMD) could also share pathological similarities through Aβ aggregation. AMD was associated with amyloid aggregation in the retinal drusen (protein-lipid deposits beneath the retinal pigment epithelium) complexes [1,18]. Patients with AMD were reported to have a significantly higher risk of developing AD [19,20,21]. Furthermore, the prevalence of AMD was more common in AD and mild cognitive impairment (MCI) patients, compared to healthy controls [22,23]. The brain-versus-eye distinction used in this review was intended as a simplified organizational framework rather than a formal disease taxonomy. The terms “brain blindness” and “eye blindness” were not established clinical classifications for visual impairment in AD. They were used to distinguish between dysfunction of central visual-processing systems and abnormalities of the retina or other ocular structures. In practice, these mechanisms could overlap, and the conditions discussed in this review, including PCA, hereditary cerebral amyloid angiopathy, glaucoma, AMD, and retinal degeneration, were clinically and pathologically distinct [1,2,3,4,5,6,7,8,9,10,11,12,13,14,15,16,17,18,19,20,21,22,23].

While there was no mutation in “classical” Alzheimer’s (AD) genes that cause immediate, complete blindness in the way a congenital retinal disease might, several specific genetic variants significantly increase the risk of severe visual impairment, including PCA or “brain blindness.” These could be categorized into mutations that cause cortical blindness (the brain cannot see) and those that link AD to retinal diseases like macular degeneration (the eye cannot see). In this review, “brain blindness” and “eye blindness” were used only as conceptual labels for central and peripheral sources of visual dysfunction. They were not formal diagnostic categories and should not be used as substitutes for PCA, PCA-AD, cortical blindness, glaucoma, AMD, or other established clinical diagnoses.

## 2. Review Methodology

This article was generally a narrative review, which was developed through a structured literature search addressing the genetic, molecular, pathological, and clinical relationships between Alzheimer’s disease and visual impairment. Searches were performed in PubMed, Web of Science, and Scopus databases. The literature search covered publications available from January 1990 to 2026 August [24].

The search strategy combined terms related to AD, visual impairment, posterior cortical atrophy, retinal dysfunction, glaucoma, age-related macular degeneration, cerebral amyloid angiopathy, apolipoprotein E, amyloid precursor protein, genetic variants, neuroinflammation, and retinal or cortical pathology. Representative search concepts included “Alzheimer’s disease” AND “visual impairment”; “posterior cortical atrophy” AND genetics; “APOE” AND retina; “APOE” AND age-related macular degeneration; “hereditary cerebral amyloid angiopathy” AND visual symptoms; “APP Dutch” OR “APP Iowa”; and “retinal degeneration” AND Alzheimer’s disease. Search terms were adapted to the indexing requirements of each database.

Studies were considered eligible when they addressed at least one of the following themes: (1) visual or retinal abnormalities in Alzheimer’s disease; (2) cortical visual dysfunction or posterior cortical atrophy; (3) genetic factors associated with Alzheimer’s disease, posterior cortical atrophy, or related visual phenotypes; (4) APP-related hereditary cerebral amyloid angiopathy and visual consequences; (5) APOE biology in the brain and retina; or (6) molecular mechanisms linking neurodegeneration, vascular pathology, retinal injury, and visual impairment. Peer-reviewed original studies, clinical series, genetic association studies, pathological investigations, and relevant narrative or critical reviews were considered when they provided information directly relevant to these themes. Studies were excluded when they were unrelated to Alzheimer’s disease or visual impairment, focused exclusively on non-human disease mechanisms without relevance to the review’s themes, lacked sufficient methodological or clinical information, or duplicated findings already represented by a more complete publication. Reference lists of key articles were examined to identify additional relevant studies, particularly for rare genetic variants, hereditary cerebral amyloid angiopathy, posterior cortical atrophy, and retinal pathology [24].

Evidence was organized into the following domains: central visual dysfunction and posterior cortical atrophy; retinal and ocular abnormalities; hereditary cerebral amyloid angiopathy and vascular visual injury; APOE-related brain–retina mechanisms; genetic associations; and potential molecular links between Alzheimer’s disease and retinal degeneration. Within each domain, greater interpretive weight was given to replicated genetic studies, well-characterized clinical cohorts, established disease-causing mutations, and studies with direct pathological or clinical evidence. Isolated case reports, exploratory loci, experimental models, and unreplicated associations were retained when relevant but were identified as preliminary studies rather than conclusive [24].

## 3. Central Visual Impairment (Brain Blindness): Genetic Basis of PCA-AD

### 3.1. PSEN1 and PSEN2: Rare Disease-Causing Mutations with PCA-AD

The genetics of PCA-AD remained unclear; no gene was described as a causative factor for it. Genetic data, reported in patients with PCA-AD, should therefore be interpreted according to their evidentiary category rather than discussed collectively. A small number of pathogenic familial AD mutations, particularly in PSEN1 and PSEN2, were associated with PCA or PCA-like clinical phenotypes [25,26,27,28,29,30]. Patients with AD and PCA could develop disease phenotypes under 65 years of age. Li et al. (2022) found no difference between sporadic EOAD and EOAD with PCA in the case of gender, disease duration, or MMSE scores [25]. No definite genetic factors were described for PCA patients [23,24,25]. EOAD patients with PCA presented similar biomarker hallmarks as typical AD, including elevated total tau (t-tau) and phospho-tau (p-tau) and reduced Aβ in cerebrospinal fluid [25,26,27,28,29,30].

PSEN1 and PSEN2 mutations presented phenotypical heterogeneity besides memory decline, other symptoms, behavioral issues, or motor impairment (Table 1). PCA was also reported in patients with PSEN1 or PSEN2 mutations [31,32,33]. The majority of PCA patients with PSEN1 or PSEN2 mutations did not have a family history of disease, but cases with family history of disease were also reported [32,33]. Patients with PSEN1 and PSEN2 mutations and PCA-AD could experience visuoperceptual and visuospatial processing and present higher-order visual dysfunction. Episodic memory could remain intact in these patients at the early disease stage. These mutations were mostly associated with early-onset symptoms (under 65 years), including simultanagnosia, optic ataxia, acalculia, agraphia, or ocular apraxia [34,35,36,37]. Even though pathological studies remained limited in these patients, histological and immunohistochemical studies on the patient (and her daughter) with PSEN1 Ile229Phe showed severe Aβ and tau deposition in occipital cortex and occipital cortex thinning [38]. Furthermore, patients with two PSEN1 mutations, Pro218Leu and Ile238Met, were associated with visual loss besides the memory dysfunctions, but PCA was not confirmed in them [39].

The pathways in which PSEN1 and PSEN2 could be involved in damage to the occipital cortex remained unclear. Structure predictions revealed that PSEN1 Ile211Met could result in impairment in the interactions between PSEN1 and PEN2. The reduced interactions between PSEN1 and PEN2 were suggested to result in favored PSEN2-related gamma secretase formation and increased Ab42/40 ratio. It may not be ruled out that in the neurons, the effects in PCA could be more vulnerable to the imbalance between PSEN1 and PSEN2 containing gamma secretases [40,41]. PSEN2 mutations, including PSEN2 Met239Thr, were associated with PCA probably through non-amyloidonergic pathways, including intracellular autophagy or impaired calcium signaling [34,42].

**Table 1:** PSEN1 and PSEN2 mutations involved in PSEN1-PSEN2.

	Mutation	AOO	Family History	Phenotype	Imaging	Biomarker	Evidence	References
PSEN1	Arg157Ser	51	NA	Memory issues and depression	MRI: PCA mild medial temporal atrophy	NA	Case study/low evidence	[43]
	Ile211Met	64	Probable negative	Progressive visual problems, visual agnosia, optic ataxia, simultagnosia, prosopagnosia, and unilateral neglect alexia	MRI: cortical and subcortical atrophy in both occipital and parietal lobes PET: bilateral hypoperfusion within the parietal, occipital, and temporal lobes	Possibly disrupt PSEN1-PEN2 interaction	Case study, structure prediction/low evidence	[41]
	Gln223Arg	37	positive, mother had similar symptoms	Gait disorder, visual agnosia, and cognitive decline	MRI: PCA PET: reduced brain metabolism	Reduced CSF-Ab, increased CSF-tau	Case study/low evidence	[44]
	Ile229Phe	47	positive, one daughter had similar phenotype	Memory, gait, and visual disturbances	MRI: atrophy in parieto-occipital lobe, frontal grey matter loss FDG-PET: bilateral parietal lobes hypometabolism PiB PET: amyloid positivity	Histology: Aβ and tau burden in occipital cortex	Case study/low evidence	[38]
	Ala434Val	52	positive	Memory impairment, abnormal visual-spatial abilities, simultanagnosia, acalculia, and agraphia	CT: posterior pattern atrophy FDG-PET: left predominant hypometabolism (occipital, parietal, and temporal regions)	Reduced CSF-Ab, increased CSF-tau	Case study/low evidence	[33]
	Pro436Gln	26	negative	Cognitive decline, anxiety, apathy, visual issues, and learning difficulties.	EEG: slow waves MRI: bilateral parieto-occipital atrophy FDG-PET: hypometabolism in parieto-occipital cortex	CSF reduced A42/40, increased p-tau	Case study/low evidence	[44,45]
PSEN2	Ser130Leu	41	Not available	Visual impairment, agnosia, intact attention, and verbal memory disturbances	Voxel spectroscopy: decreased NAA in right parieto-occipital white matter FDG-PET: decreased metabolism	NA	Case study/low evidence	[27]
	Met239Thr	59	negative	Memory decline, agnosia, right-left confusion, and impaired episodic memory. Simultanagnosia and prosopagnosis	MRI: Bilateral hippocampi and temporal atrophy PET: extensive Aβ deposition	NA	Case study/low evidence	[32,42]
	Val363Ile	52	positive	Visual issues, depression, and memory issues	FDG-PET: biparietal and right frontotemporal hypometabolism.	Reduced CSF-Aβ increased p-tau and t-tau	Case study	[46]

### 3.2. Other AD Risk Factor and Candidate Genes in PCA-AD

Studies were conducted on AD risk genes and PCA, and several of them impacted visual impairments, including APOE E4, CLU, ABCA7, or PICALM. Some results suggested a possible susceptibility effect, but none of them suggested deterministic causation. These results should be replicated in independent cohorts with a larger number of patients. Other observations derived from isolated reports or a small number of patients (including GRN, MAPT, and TREM2). Such reports identified clinically relevant possibilities, but they did not establish that the reported variants could be involved in PCA or predict the syndrome. Accordingly, the current evidence supported heterogeneous and incompletely defined genetic contributions, rather than a single established genetic explanation for PCA [47,48,49,50,51,52,53,54,55,56].

Among the AD risk factors, APOE E4 allele was suggested to be risk for both posterior AD and PCA. APOE E4 was associated with increased PCA risk with an odds ratio of 3.17 and *p* = 0.0003. Age of onset in the case of PCA and posterior AD was earlier in the case of APOE E4 allele, compared to E3 and E2 carriers. No other variant reached the same level of significance; different intronic variants were reported to impact PCA and posterior AD, including protective variants in CLU, and a risk variant in BIN1. The rs3764650 in ABCA7 could also increase the risk for PCA in a homozygous stage, but further confirmation should be needed. The PICALM rs3851179 variant showed strong association with clinical PCA, but not in posterior AD [55]. Another study screened AD risk variants and association with PCA. This study confirmed the significant association between PCA and APOE E4, ABCA7 rs3764650, and BIN1 rs744373. Also, the same study reported additional putative PCA-associated variants, including rs2075650 in TOMM40, rs3818361 in CR1, or rs76854344 near CNTNAP5 [56]. Exploratory GWAS findings near SEMA3C, CNTNAP5, and FAM46A should be defined as potential candidate loci for AD and PCA. However, independent replication should be needed, rather than as established PCA genes [56].

Evidence involving TREM2 was based primarily on rare-variant screening rather than on a replicated PCA-specific association. The AD risk factor Arg47His variant in TREM2 was suggested to play a role in atypical AD phenotypes [25,51]. Patients with this variant were associated with AD [27,52,53,54,55]. Brain pathology study on one of the PCA patients confirmed the presence of occipital lobe atrophy, and advanced AD pathology (amyloid plaques and severe neurofibrillary tangles) was present in the posterior brain region. However, TREM2 Arg47His was commonly described in typical AD pathology, suggesting that this variant was not directly linked to PCA [53]. It may not be ruled out that TREM2 Arg47His could interact with other putative genetic and environmental factors, which could increase the vulnerability of posterior regions in patients [27,53].

The PCA was found to be an atypical phenotype in the case of frontotemporal dementia-associated granulin (GRN) mutations. A GRN patient with the nonsense variant, GRN Arg110Ter, presented abnormalities in basic visuoperceptual skills, including apperceptive visual agnosia, alexia, and prosopagnosia. Granulin levels were reduced in patients’ plasma [47]. Another patient with GRN Thr227Serfs presented abnormal visuospatial skills, including dyscalculia, simultanagnosia, optic ataxia, or oculomotor apraxia. Imaging presented atrophy in the right parieto-occipital cortices. The CSF biomarkers ruled out the AD diagnosis [48]. Further studies should be needed, through which pathways GRN could contribute to PCA. However, the role of GRN haploinsufficiency could result in diverse phenotypes, and it was also suggested to be involved in AD pathology. These studies suggested that GRN haploinsufficiency could possibly affect PCA [48]. The common intronic rs5848 was also analyzed in corticobasal syndrome and PCA, and its homozygous TT allele was a risk factor for both PCA and CBS [49,50]. MAPT Val363Ile mutation was found in a patient with corticobasal syndrome, progressive supranuclear palsy, and PCA at the age of 49 years. MRI revealed atrophy in the mesencephalon and right parieto-occipital region, while FDG-PET showed hypometabolism in different brain areas, including the right frontal, parietal, and occipital lobes and right striatum. AD pathology was excluded [51]. Another MAPT mutation, Pro354Leu, was found in a patient with atypical AD with PCA. This patient was tested with a [18F]PI2620 tau PET, which showed increased tracer uptake in different brain areas, including temporal, parietal, and occipital lobes, suggesting that he had high tau positivity. Amyloid PET was also positive [52]. Reports involving GRN and MAPT should be presented as individual or limited observations that broaden the range of possible PCA-like phenotypes.

## 4. APP Mutations and “Vascular Blindness”

APP mutations, including Glu693Gln (Dutch APP) and Asp694Asn (Iowa APP), were discussed primarily in the context of hereditary cerebral amyloid angiopathy (H-CAA) rather than as specific genetic causes of AD-related visual dysfunction. These mutations were found to promote vascular amyloid deposition and resulted in vascular complications, including cerebral hemorrhage, occipital calcification, and damage to posterior visual pathway [57]. The visual impairment in affected individuals was caused by vascular injury involving occipital or other visual-processing regions. This mechanism should be distinguished from PCA, in which visual symptoms reflect neurodegenerative dysfunction of cortical visual networks. Although the Dutch and Iowa variants caused dementia with AD-like features, their vascular manifestations should not be interpreted as evidence of a distinct AD visual phenotype [57,58,59,60,61,62].

The Dutch APP associated variant was characterized by intracerebral hemorrhages, which often affected the occipital lobes. These recurrent vascular changes could result in visual dysfunctions or even the complete loss of vision [58,59,60]. Occipital cortical calcifications were present in the case of Dutch APP mutation, which could be predictors of intracerebral hemorrhage (ICH) in the pre-symptomatic carriers [61]. The Iowa APP was found in a family from Iowa, and affected relatives presented progressive aphasic dementia, leukoencephalopathy, and calcifications in occipital brain areas. Patients also presented visual dysfunctions [62]. Their neuropathy showed severe CAA, neurofibrillary tangles, and extensive Aβ40 distribution inside the plaques. The age of onset was between 48–53 years. The non-carrier relatives were cognitively normal and did not present any cerebral hemorrhage [62]. Another patient with Iowa APP developed migraine with visual aura and language and memory issues and was diagnosed with hereditary cerebral amyloid angiopathy (HCAA) in his thirties. Vascular issues (ischemic or hemorrhagic stroke and calcifications) appeared at the age of 42 [63].

**Figure 1:**
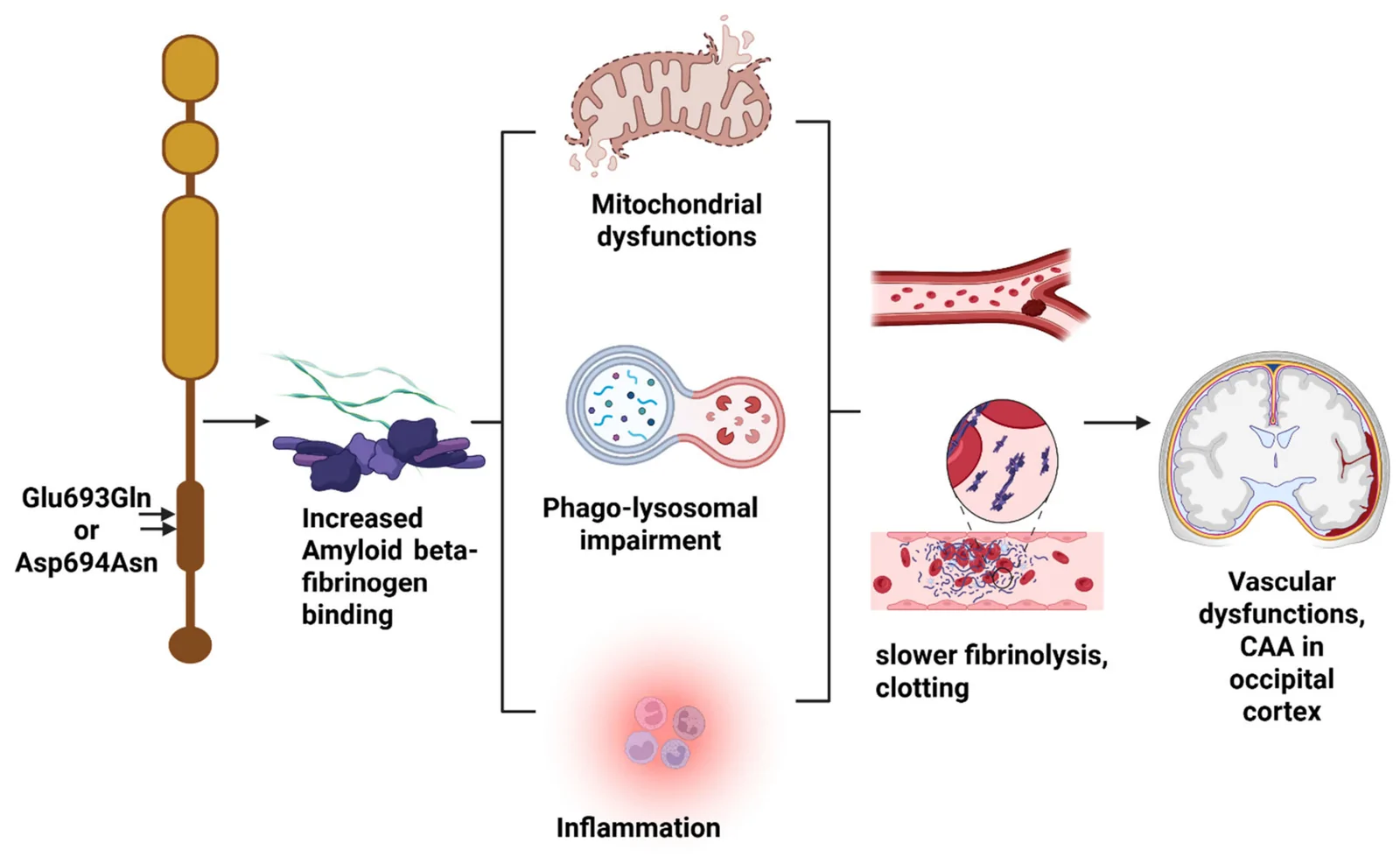
Impact of Iowa APP and Dutch APP on the brain vascular system. These mutations increased the Aβ–fibrinogen interaction, resulting in mitochondrial dysfunctions, inflammation, phago-lysosomal impairment, and vascular dysfunctions, resulting in CAA. Created in BioRender. An, S. S. A. (2026) https://BioRender.com/3y2ofqz., accessed on 10 September 2026.

In case of both Dutch and Iowa APP, HCAA was verified as the common disease phenotype, where Aβ could be deposited in vascular system. These mutations were associated with increased degrees (50-fold) of Aβ binding to fibrinogen. The interaction between fibrinogen and mutant APP could also result in a higher tendency for clotting and slower fibrinolysis [64]. Transcriptome analysis in frontal and occipital cortex of patients with Dutch APP revealed that the pathways associated with ATP synthesis and carbon metabolism were down-regulated, suggesting the presence of mitochondrial dysfunctions and lysosomal-phagosome impairment. Furthermore, additional pathways, including extracellular matrix (ECM)–receptor interaction and ECM proteoglycans were up-regulated, suggesting that the transforming growth factor beta (TGFβ) pathway was a signal of inflammatory pathways [65]. Figure 1 summarized the potential impact of Iowa APP and Dutch APP on the brain vascular system, which could result in visual dysfunctions in the patients.

## 5. APOE Isoforms and Retinal Health

Apolipoprotein E (APOE) has three main isoforms, APOE E2, E3, and E4, which could be determined by two variants, called rs7412 and rs429358. Among them, APOE E4 allele was verified to be the main risk factor for late-onset AD. However, it was reported that the APOE E4 allele lowered the risk for age-related macular degeneration (AMD). Although APOE E2 was associated with a lower risk of AD in several studies, its effects could vary by population and clinical context [66,67]. APOE E2 was also suggested to be implicated as a possible risk factor for age-AMD, which proposed the tissue-specific effects of APOE isoforms. In the Rotterdam Study, APOE E2 was associated with a slightly increased risk of AMD, whereas APOE E4 was associated with reduced risk, with an odds ratio of 0.43 [68,69,70,71,72,73]. An earlier HuGE meta-analysis estimated a protective effect of up to 40% for APOE E4 and a risk effect of up to 20% for APOE E2. This study also suggested that survivor bias should not be definitively excluded [73]. A pooled analysis of 15 studies reported an odds ratio of 0.72 per APOE E4 haplotype for late AMD and an odds ratio of 1.83 for APOE E2 homozygotes [74]. Studies on the Copenhagen City Heart Study (CCHS) and the Copenhagen General Population Study (CGPS) cohorts revealed the reduced AMD risk for E4/4 and E4/3 genotypes and higher risk for E3/2 than E3/3 [75].

A possible explanation may be associated with the altered lipid handling by APOE E2 in the retinal pigment epithelium. Experimental data indicated that APOE E2-expressing retinal pigment epithelial cells could have deficient lipid transport, contributing to lipid-rich drusen formation and impaired cellular function [67,68,69,70,71,72,73,74,75,76].

Due to the lack of reactive cysteines, APOE E4 (arginine residues at position 112 and 158) was found to be resistant to thiol-mediated phase transition and drusen formation [72]. The drusen was defined as the hallmark of extracellular deposits of AMD. APOE protein was synthesized in the retinal pigment epithelium (RPE) and Mueller cells and could play a role in trafficking and the efflux of these lipids [77,78,79,80]. The association of APOE with AMD was different across global populations due to variations in allele frequencies, genetic backgrounds, and local ancestry haplotypes. A meta-analysis reported a significant association between the APOE E2 allele and early AMD among Black and East Asian populations; however, the small sample size was a limitation of this study. It suggested that APOE E4 was generally protective against early-onset AMD [81]. Similarly, the Rotterdam Study reported that the APOE E4 allele was associated with decreased AMD risk with an odds ratio of 0.43, and it increased slightly the risk for AMD in E2 allele [66].

APOE E2 allele was found to reduce the binding affinity to the low-density lipoprotein receptor (LDLR) and increase the levels of atherogenic lipoproteins [77,78,79,80,82]. The reduced interaction between APOE and LDLR could result in lower degrees of cholesterol transport, endocytosis, clearance, and recycling [77,78,79,80,82]. The reduced cholesterol transport and clearance could result in accumulation of cholesterol and neutral lipids in the RPE and drusen formation [83]. The abnormal cholesterol homeostasis by APOE E2 could result in impaired autophagosome trafficking and lysosomal function in the RPE [84]. The RPE cells burdened by this dysregulated lipid profile could be highly vulnerable to the alternative complement system. Complement attacks could induce mitochondrial fragmentation and cell stress, accelerating the progression of AMD pathology [84,85]. The APOE E2 allele overexpression in the mononuclear phagocytes (MP) due to aging and stress could induce pro-inflammatory mechanisms, including the TLR2-CD14 innate immunity receptor cluster. This pathway could promote the release of pro-inflammatory molecules, including IL-6 or CCL-2 [82,86]. APOE4 is suggested to protect against subretinal inflammatory pathways, since in APOE E4 (TRE4) mice, reduced levels of APOE and CCL2 prevented the abnormal MP accumulation in the retina [82].

Controversially, the APOE E4 expression could prevent the pathological accumulation of cholesterol in the RPE, even in the presence of stressors, including the lipofuscin bisretinoids [82]. The APOE E4 could bind with high affinity to LDLR receptors, which could induce lipid recycling [69]. Also, APOE E4-containing vesicles were reported to have long-range, directed movements, which could allow the efficient cholesterol and neural lipid clearance [72]. In the case of lipid stress, APOE E4-expressing cells could express neutral lipids in the lipid droplets, which protected against oxidative stress in RPE [87]. Taken together, the APOE alleles presented divergent associations across tissues, which was suggested as the APOE paradox. These results supported the importance of considering tissue-specific APOE biology, but they did not establish that one APOE isoform could be therapeutically substituted for another without risk. The effects of APOE-directed interventions on retinal health remain unresolved and should be assessed in preclinical models and appropriately designed clinical studies [79,80,81,83,84,85,86,87].

**Figure 2:**
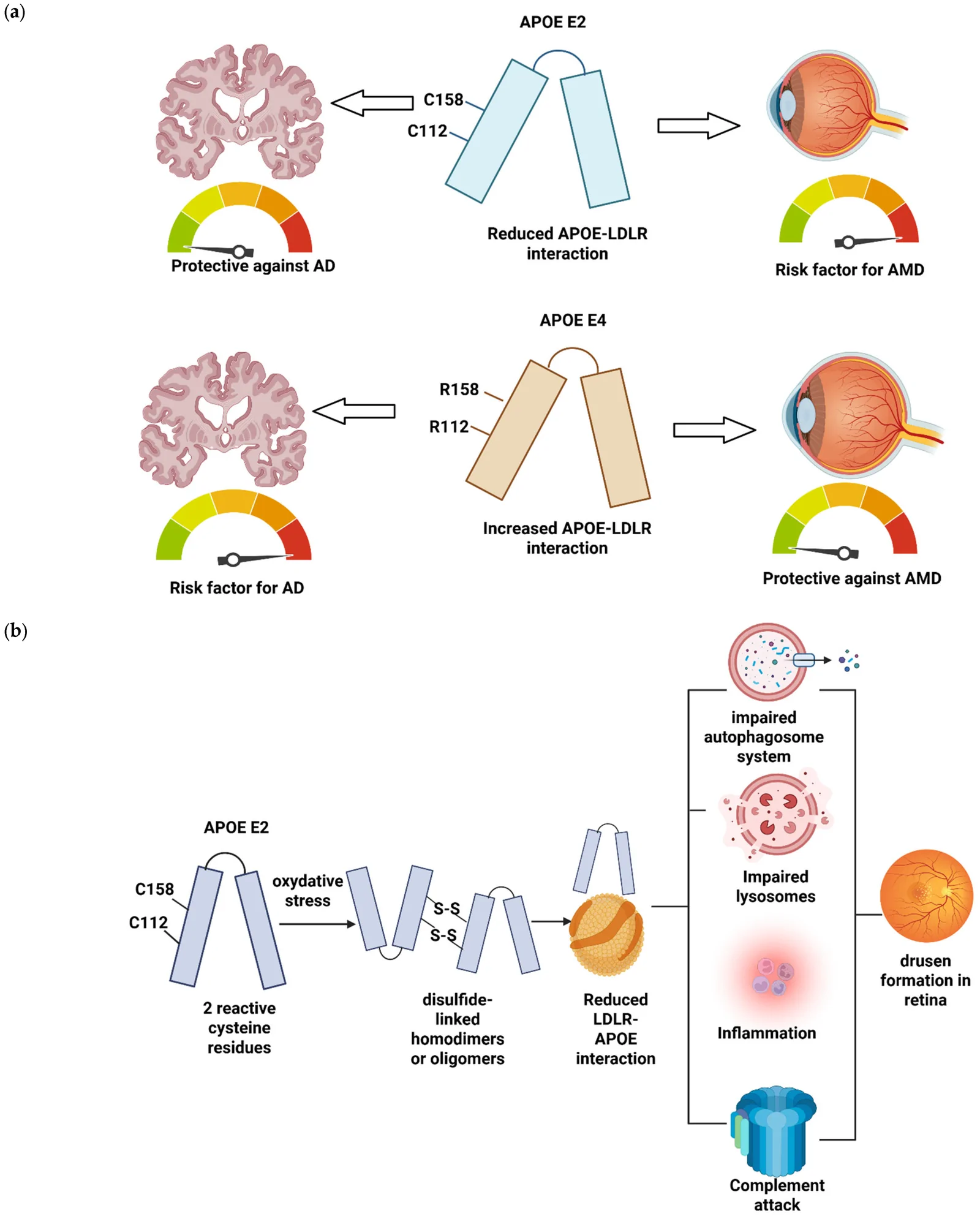
(**a**) The APOE paradox in Alzheimer’s disease and age-related macular degeneration. Schematic illustration of the opposing or potentially tissue-specific effects of APOE alleles in the brain and retina. (**b**) Possible pathways by which the APOE E2 allele influenced retinal dysfunction and drusen formation. The proposed mechanisms were hypothetical and require further validation. Created in BioRender. An, S. S. A. (2026) https://BioRender.com/ylucosk., accessed on 10 September 2026.

Figure 2b summarizes the APOE paradox. Figure 2a summarizes the basics of the APOE paradox, while Figure 2b shows the impact of the APOE E2 allele on retinal dysfunctions.

## 6. CST3 Mutation and the Risk for AMD

CST3 gene encodes cystatin C, which should be crucial for inhibiting cysteine proteases and protecting the proteins from degradation. Cystatin C was found to be an effective biomarker for kidney function, but it was expressed in most body parts. The Ala25Thr variant (rs1064039) was suggested to be risk factor for both AMD and AD in an autosomal recessive manner (B/B genotype) [88]. In the Caucasian population, the B/B genotype was more common in AMD patients, compared to controls. This allele could contribute to disease risk in German AMD patients (up to 6.6%). Association of B/B allele with AMD was stronger in males, compared to females, but disease-free survival time was similar in male and female patients [89]. In the case of AD, Caucasian studies found association between AD and B/B allele, but they failed to find this association in Asian populations [90,91,92]. The co-existence of the APOE E4 allele and CST3 B/B allele could increase the risk for late-onset AD significantly (14-fold and 16-fold in males and females, respectively). Also, the combination of CST3 B/B allele and APOE E4 allele could result in earlier disease onset [93,94,95]. This “variant B” reduced the hydrophobicity of the signal sequence, resulting in inefficient cleavage and lower secretion of cystatin C into the CSF and plasma [96]. Normally, cystatin C plays a role in neuroprotection by binding to soluble Aβ and preventing its oligomerization and fibril formation. Reduced cystatin c expression resulted in loss of neuroprotection and accumulation of Aβ in the brain and brain vessels (CAA) [97].

Research indicated that patients with AMD had approximately 48% increased risk for developing AD compared to those without AMD, suggesting there may be a common pathway between AD and AMD through reduced cystatin C levels and protein homeostasis impairment [20]. The CST3 Ala25Thr mutation resulted in disturbances in the CST3 protein, since Ala25 was a crucial residue in cystatin C, playing a role in the protein transfer to the Golgi apparatus. Individuals with B/B genotype experienced reduced cystatin C secretion (approximately 50%) and insufficient supply of CST3 protein to extracellular space [98,99]. The reduced secretion of cystatin C impaired the cathepsins regulation, which should be crucial for extracellular matrix remodeling and the release of anti-angiogenic factors [98]. The imbalance of cystatin C and cathepsins increased the degradation of Bruch’s membrane and induced angiogenesis, contributing to the development of exudative AMD [98,99]. The CST3 B/B genotype resulted in damage to RPE through mitochondrial dysfunctions. The mutant cystatin C was associated with mitochondria, rather than them being targeted to ER/Golgi pathway. In mitochondria, cystatin C interacted with different outer membrane proteins, including 18 kDa translocator protein (TSPO) and cytochrome B5 type B [98,99,100,101]. The abnormally localized cystatin C and aberrant protein interactions increased the membrane potential and could cause mitochondria to be in a hyperpolarized state. Also, the mutation increased the ROS production in the presence of stressors [99,102,103]. As mentioned before, deficient cystatin C levels reduced the anti-amyloidogenic effects and facilitated the deposition of Aβ in both the senile plaques of the brain and the drusen of the retina [104]. The combination of secretory failure and mitochondrial dysfunctions could disturb and accelerate the RPE degeneration by impairing the lysosomal clearance pathways and formation of lipofuscin. RPE cells with the B/B allele were more sensitive to age-related stresses, including the accumulation of advanced glycation end products (AGEs) in the Bruch’s membrane [99,105].

## 7. AD-Causing and Risk Genes and Glaucoma

Genome-wide association studies have identified overlapping genetic loci associated with AD and POAG. These findings suggested shared genetic susceptibility and potentially convergent biological pathways, but they did not establish direct communication or signaling between the brain and eye [106,107,108,109,110,111,112,113,114,115,116,117,118,119,120,121,122,123,124,125,126,127,128,129,130,131,132,133,134,135,136,137,138,139,140,141]. Contradictory results were reported on the role of the APOE E4 allele in POAG among different populations. In European populations (British and Finnish cohorts), the APOE E4 was suggested to protect against all forms of glaucoma, including POAG or normal tension glaucoma (NTG) [106,107]. However, studies from Asian populations revealed that the homozygous E4 allele increased the risk for POAG [108,109]. Initial smaller studies suggested that the ε4 allele was a risk factor for POAG in Saudi patients [110]. However, larger investigations in Saudi populations failed to find any significant association between common APOE variants and POAG risk [111]. APOE E4 carriers experienced quicker thinning of macular ganglion cell-inner plexiform layer (mGCIPL) complex and the peripapillary retinal nerve fiber layer (pRNFL), which could induce the ganglion cell degeneration in glaucoma [112]. Studies on glaucoma mouse models suggested that APOE4 protected against retinal ganglion cell loss by preventing microglia from transitioning into a neurodegenerative phenotype, even in the case where the intraocular pressure was elevated. In the case of neurodegeneration, Galectin-3 was upregulated. However, APOE E4-expressing microglia, the galectin-3 upregulation, could be prevented, resulting in extended survival of neurons in the retina [106,113].

Even though APP mutations were associated with glaucoma, APP was found to play a central role in glaucoma onset [114]. Even though APP was not necessary for the basic vision, it was suggested to play a crucial role in the inner retinal functions. APP was expressed in the inner nuclear layer (INL) and the ganglion cell layer (GCL) of the retina. APP knockout mice showed impairment in rod and cone pathways, including reduced b-wave amplitude [115]. In both the brain and retina, normally, APP was cleaved by the alpha secretase. In the case of increased intraocular pressure, caspase-3 was activated, resulting in abnormal APP cleavage, called delta-C APP. Also, caspase-8 induced the mitochondrial pathways through Bid cleavage, which could accelerate the caspase-3 activation and APP processing [17,116]. Increased levels of deltaC-APP were elevated in retinal ganglion cells (RGC), which could be neurotoxic [118].

APP was cleaved by β- and γ-secretases in the retina, and chronic elevation of intraocular pressure was associated with increased Aβ generations, retinal Aβ accumulations, inflammatory signaling, and retinal ganglion cell toxicity [119,120,121]. However, evidence from AD patients and 5 × FAD mice indicated that retinal Aβ also originated from the brain. Aβ was transported through the cerebrospinal fluid along the optic nerve and entered the retina through perivascular and axonal pathways, while AQP4 induced this brain-to-eye transport and subsequent ocular clearance [122]. In AD patients and 5 × FAD mice, Aβ deposits were detected in the ocular glymphatic system, optic nerve, retinal vessels, and perivascular spaces, whereas retinal APP and PS1 expressions were not significantly increased, suggesting that elevated retinal Aβ was not explained solely by local production, but a possible brain-to-eye pathway. The optic nerve and its associated CSF and perivascular pathways could provide a structural route through which brain-derived pathological proteins reached the retina, linking cerebral amyloid pathology with retinal degeneration. In this theoretical framework, the retina may reflect not only locally generated pathological changes, but also the movement and clearance of neurotoxic substances originating in the brain. However, further studies were needed to verify the clinical significance and directionality of this pathway in human studies [122].

MAPT variants were verified to play a role in both neurodegenerative diseases (AD and FTD) and glaucoma. Particularly, an intronic variant, rs242559, was suggested to be a risk variant for both AD and POAG [123,124,125,126,127]. MAPT variants were associated with changes in retinal morphology, including the thinning of the macular retinal nerve fiber layer (RNFL) and the ganglion cell-inner plexiform layer (GCIPL) [128,129]. MAPT dysfunctions were not associated with intraocular pressure (IOP), but they were suggested to impact glaucoma through pressure-independent mechanisms [130,131]. Since tau could play a role in microtubule stability, abnormal tau expression was verified to impact glaucoma onset [132]. Deficient microtubules and cytoskeleton could result in disruption of RGCs [132,133]. In retina, aggregation of hyperphosphorylated tau and neurofibrillary tangles were observed in the retina, which could contribute to synaptic dysfunctions and impaired axonal transport system [133,134,135]. The impaired axonal transport could result in impaired delivery of brain-derived neurotrophic factor (BDNF), which increased the excitotoxic sensitivity of the RGC and reduced RGC survival [136,137]. Tau impairment could also inhibit transport of mitochondria and peroxisomes through the kinesin-dependent pathways, resulting in energy dysfunctions and oxidative stress [137]. Tau dysfunctions could activate the programmed cell death pathways in glaucoma, including caspase-2 and caspase-3 enzymes, which could induce RGC death [138]. Also, abnormal tau phosphorylation promoted the unfolded protein response, endoplasmic reticulum stress, and neuroinflammation, resulting in severe damage to the retina [139].

Genome-wide association studies (GWAS) found a significant overlap between POAG and AD and several potential candidate variants, located on chromosome 11. The meta-analysis identified 49 loci, including at least 11 protein-coding genes, which could be risk factors for both POAG and AD. They were considered as “shared genetic architecture,” and they could impact the progression and development of these two diseases [132,140]. Functional genomic analyses revealed that these genes modulated activities inside the mitochondria, nucleus, or cellular membranes. For example, MTCH2, NDUFS3, or PTPMT1 genes were located on the inner mitochondrial membrane and impact roles in mitochondrial metabolism. Network analyses further suggested that these genes, along with the SPI1 and MYBPC3, may function consistently, and alteration in their expression levels could be associated metabolic dysfunction and pathogenic processes in the brain and retina [132,140,141]. These studies proposed that there could be a putative “inter-organ crosstalk” between the brain and the eye [140]. The SPI1 gene was verified to play a role in macrophage activation, which was critical for the microglial response to Aβ plaques in AD and inflammatory damage in the brain and retina. Additionally, the RAPSN and CELF1 were found to regulate cell death and apoptotic signaling, contributing to progressive irreversible cell loss in both AD and glaucoma [140]. Chromosome 11 represented an important genomic region linking POAG and AD because several shared loci and genes associated with these diseases were reported in this region. Their involvement in pathways related to mitochondrial function and immune regulation provided a possible functional basis for common processes affecting the brain and retina, although the specific contribution of chromosome 11 to the shared genetic variants between AD and glaucoma required further investigation. However, genetic overlap alone did not demonstrate functional communication between the brain and the eye. These data could reflect the pleiotropy, shared susceptibility mechanisms, or related cellular processes. The possibility of shared genetic architecture between brain and ocular disease remained a hypothesis that required direct mechanistic investigation. Shared loci and pathway enrichment indicated overlapping genetic architecture, but they did not demonstrate tissue-to-tissue signaling, anatomical communication, or a causal relationship between AD and glaucoma by themselves. Understanding the common pathways between these two diseases could be useful to develop treatment strategies for both AD and POAG [132,140,141,142]. Table 2 summarizes the genes involved in both AD and glaucoma.

## 8. Discussion

Visual impairment in Alzheimer’s disease (AD) could have a dual-facet paradigm: central “brain blindness,” caused by neurodegeneration in posterior cortices, or peripheral “ocular blindness,” driven by localized microenvironmental changes in the retina [53,143]. Distinguishing central processing deficits from peripheral structural pathology was clinically essential but remained diagnostically challenging due to overlapping presentations [53,142,143]. A major source of diagnostic delay was possible in the case of PCA, marked by prominent higher-order visual deficits, including simultanagnosia, optic ataxia, and visual agnosia, that regularly appeared years before the memory loss [31,34,143,144,145,146,147]. Because initial complaints were predominantly visual, patients routinely present to eye care professionals, resulting in the misdiagnosis as primary ocular disorders and subsequent treatment delays [145,146]. Neuro-ophthalmologists must focus on identifying PCA in older adults with unexplained visual signs, since traditional vision tests failed to capture higher-order visual dysfunctions [14,55,145,148,149].

However, the diagnostic utility of genetics in visual-variant AD remained to be confounded by biological and methodological complexities, including etiological heterogeneity, vascular versus amyloid specificity, or shared pathophysiology with glaucoma. In the case of visual-variant AD, while neurofibrillary tangles and senile plaques accumulated in the occipitoparietal and occipitotemporal visual processing regions in classical PCA [33,36,143,147], clinical PCA was not pathologically uniform. In addition, PCA should be regarded primarily as a clinical-radiological syndrome defined by progressive impairment of higher-order visual processing and characteristic posterior cortical involvement, rather than as a synonym for AD [7]. PCA due to AD pathology (PCA-AD) referred to patients who met clinical criteria for PCA and in whom Alzheimer’s disease was established or considered the primary underlying pathology. Although AD was found to be the most common pathology associated with PCA, the underlying pathology could be variable. Non-AD PCA or PCA-like phenotypes should describe similar clinical presentations associated with another pathology or with uncertain etiology. Cortical blindness is distinct from PCA because it denoted severe visual loss from the dysfunction of the posterior visual cortex or visual pathways, not a specific neurodegenerative syndrome [7,150]. Up to 20–30% of PCA presentations were driven by non-AD pathologies, including Lewy body disease, corticobasal degeneration, or prion disease [150,151]. Existing genetic association studies routinely failed to stratify patients by biomarker-verified etiology, obscuring true locus-specific risks [151,152]. In the case of vascular versus amyloid specificity, the specific mutations in APP (e.g., Dutch and Iowa variants) increased the binding affinity of mutant Aβ to fibrinogen, resulting in cerebrovascular fibrin deposition, microbleeds, occipital hemorrhages, and “vascular blindness” [64,152,153]. Recognizing the fibrinogen–amyloid axis would provide a diagnostic marker for vascular dysfunction [152,153]; however, it remained unclear whether milder vascular alterations in sporadic AD induced the visual loss independently or acted synergistically with neurodegeneration [152,153,154]. In the case of shared pathophysiology with glaucoma, epidemiological and GWAS data revealed significant overlap between AD and glaucoma, including at least 49 shared genomic loci (including MTCH2, NDUFS3, or PTPMT1) and retinal accumulations of Aβ and hyperphosphorylated tau [140,155,156,157]. Chromosome 11 represented an important genomic region linking POAG and AD because several shared loci and genes associated with these diseases were reported in this region. Their involvement in pathways related to mitochondrial function and immune regulation provided a possible functional basis for common processes affecting the brain and retina, although the specific contribution of chromosome 11 to the shared genetic variants between AD and glaucoma required further investigation. However, genetic overlap alone did not demonstrate functional communication between the brain and the eye. These data could reflect the pleiotropy, shared susceptibility mechanisms, or related cellular processes. The possibility of shared genetic architecture between brain and ocular disease remained a hypothesis that required direct mechanistic investigation. Shared loci and pathway enrichment indicated overlapping genetic architecture, but they did not demonstrate tissue-to-tissue signaling, anatomical communication, or a causal relationship between AD and glaucoma by themselves. Understanding the common pathways between these two diseases could be useful to develop treatment strategies for both AD and POAG [132,140,141,158].

Translating genetic and mechanistic data into potential treatments could have several pharmacological and targeting obstacles, including the APOE paradox. APOE E2 was associated with relative protection against AD, but a potential risk for AMD [72,159]. Experimental studies suggested that altered APOE-dependent lipid handling may affect RPE cholesterol homeostasis, cellular stress, inflammation, and drusen-related processes. These data provided a possible mechanism for the observed APOE–AMD associations, but they did not establish a complete causal pathway in human AMD [71,88,160,161]. In contrast, APOE E4 was found to be the strongest risk factor for AD, which was associated with reduced Aβ clearance and increased Ab aggregation and deposition. However, APOE E4 was found to be protective against AMD in the retina [71,159]. This divergent role reflected a complex evolutionary compromise between lipid homeostasis and immune regulation [160,161]. In the retina, experimental studies suggested that APOE isoforms may differentially affect lipid transport, RPE homeostasis, and subretinal inflammation [64,162]. At present, the available evidence may not establish that reducing brain APOE E4 could increase AMD risk or that APOE E2-mimicking therapies cause retinal inflammation or microvascular dysfunction. These possibilities remained as hypotheses requiring direct evaluation in retinal models and clinical studies. APOE-directed interventions should be performed separately for neurological and retinal effects because the data from AD patients may not be directly extrapolated to AMD patients [2,64,161,162,163,164]. Overcoming this paradoxical relationship would require strict tissue and cell-type targeting [165,166,167,168]. While viral vectors with brain-specific promoters and BBB-transporter delivery systems were currently under active development, further studies should be needed on their ability to prevent off-target retinal toxicity in human trials [165,166,167,168]. In cases of vascular blindness driven by Iowa or Dutch APP mutations, inhibiting the Aβ-fibrinogen interaction could be a targeted strategy to protect against both vascular and cognitive decline [169,170]. However, advanced delivery platforms, including liposomes and nanotherapeutics, failed to achieve selective therapeutic levels in posterior visual hubs without off-target effects [149,169,170,171,172,173]. Furthermore, in pure PCA, primary ocular interventions were not beneficial for central processing deficits, underscoring the requirement for precise anatomical targeting [33,171].

There were several limitations to the study of AD-causing and risk genes and visual impairment. First, limited genetic evidence was identified for PCA. Despite reported associations of variants in PSEN1, PSEN2, BIN1, and PICALM with PCA or atypical presentations [31,55,144,146], the available data failed to establish any of these variants as specific or sufficient predictors of PCA [54,151]. The majority of genetic observations came from isolated case reports or small AD cohorts [56,151]. Future studies should be conducted on larger, prospectively recruited cohorts with standardized clinical criteria, longitudinal tracking, and biomarker-supported etiological classification [151,152]. Furthermore, there was a population bias in genetic datasets. Most AD-GWAS data originated from cohorts of European ancestry [174]. Because allele frequencies and genomic architectures could be different across populations, current risk-prediction models lack global generalizability [174]. Visual impairment and AD genetic research should focus on multi-ancestry recruitment and independent replication to validate identified loci [174]. An additional issue could be that the current mechanistic data relied heavily on cell cultures, organoid models, and transgenic animals (including APOE4 mice) [163]. Rodent eyes were significantly different from human eyes, including the lack of true anatomical macula, and the RPE lipid transport and complement activation pathways could be different in them, limiting the direct translational value of these models [163]. Finally, the retinal imaging did not have a strong diagnostic value. Although non-invasive retinal thickness and imaging metrics correlated with AD and PCA features [175,176], current studies were cross-sectional and failed to show whether retinal alterations could possess sufficient specificity to serve as potential diagnostic biomarkers by themselves. Prospective longitudinal studies should be essential to establish the temporal sequence and diagnostic accuracy of retinal biomarkers relative to the central pathology [149,173,175,176,177].

Taken together, genetic profiling of patients would be crucial for personalized medicine in AD, since mutations in different genes (e.g., PSEN1, PSEN2, or CST3) showed unique phenotypes, including early visuospatial dysfunction or aggressive cognitive decline [30,88,144,178,179]. Genetic data involving CST3 and other risk variants should therefore be interpreted cautiously and distinguished from established disease-causing mutations. Because visual-system involvement occurred early in some patients, integrating genetic information with longitudinal cognitive, visuospatial, and ophthalmic assessments could improve the identification of individuals at risk for atypical presentation and help clarify disease progression. However, visual disturbances should be considered as a potential, rather than definitive, predictor of AD onset because current evidence showed that early visual involvement remained inconclusive. Further validation in larger, diverse, and well-characterized cohorts would be needed before genetic and visual markers could be reliably incorporated into clinical prediction and personalized treatment strategies.

## Figures and Tables

**Table 2:** Genes that could be involved in both AD and POAG [98,99,100,101,102,103,104,105,106,107,108,109,110,111,112,113,114,115,116,117,118,119,120,121,122,123,124,125,126,127,128,129,130,131,132].

Gene	Variants	Function	Impact in AD	Impact on POAG	Evidence
APOE	E4 allele	Lipid transport, Aβ clearance	E4 allele main risk for AD, reduced	Contradictory studies, either risk or protective	Association studies/low to moderate
APP	abnormal expression	Aβ precursor	Increased degree generation of Aβ generation	Possible impact on retinal functions, Aβ deposits in retina	Functional studies/moderate
MAPT	rs242559	Cytoskeletal protein	Abnormal tau phosphorylation, neurofibrillary tangle formation	Tau impairment and tangle, reduced RGC survival	Functional studies/moderate
AGBL2 (ATP/GTP-binding protein-like 2)	rs11604825 rs11602395	Impacts alpha tubulin post-translational modification	Abnormal axonal transport	Increased IOP	Association studies/moderate
SPI1 (Spi-1 proto-oncogene)	At least 28 variants, including rs11039199 or rs7105851	Transcription factor regulates immune cell development and hematopoiesis	Microglial activation apoptosis, immune functions	Microglial activation apoptosis, immune functions, increased IOP	Association studies/moderate
CELF1 (CUG triplet repeat, RNA binding protein 1 Elav-like family member 1)	rs4752845 rs34958982 rs66749409 rs12798346 rs56400411	Gene expression regulator at post-transcriptional level: controls alternative splicing, mRNA stability	Impacts AD through tau splicing. Possible impact on apoptosis pathways	Apoptosis pathways and increased IOP	Association studies/moderate
FAM180B (Family with sequence similarity 180 member B)	rs11605348	Unknown, but expressed in fat tissues, skin and the thyroid gland, possible play a role in immune response	Putative role in Aβ processing, increased levels in AD brain, probably neuroinflammation	Increased IOP and neuroinflammation	Association studies/moderate
MTCH2 (Mitochondrial carrier 2)	rs4752856 rs4752856 rs4752856	Mitochondrial membrane protein, regulates apoptosis, energy homeostasis, and survival of cells	Impaired mitochondrial metabolism and energy transport	Increased IOP, abnormal mitochondrial metabolism and apoptosis	Association studies/moderate
MYBPC3 (Myosin binding protein C3)	rs2856661	Impacts the heart muscle cell functions and development	Putative effect on axonal growth and development	Increased IOP	Association studies/moderate
NDUFS3 (NADH: ubiquinone oxidoreductase core subunit S3)	rs2030166 rs2030166	Mitochondrial complex subunit, could impact energy production, aerobic respiration, and complex assembly	Impaired mitochondrial pathways, abnormal endocytic pathway, Aβ, and tau accumulation	Retinal cell death through reduced energy metabolism	Association studies/moderate
PSMC3 (Proteasome 26S subunit, ATPase 3)	rs11600581	Part of proteosome system, cellular quality control	Aβ clearance, prevents its aggregation	PSMC impairment possibly resulted in proteotoxic stress	Association studies/moderate
PTPMT1 (Phosphatidyl-glycerophosphatase and protein-tyrosine phosphatase 1)	rs56400411 rs7945473	Mitochondrial lipid phosphatase, regulation of mitochondrial energy-flexibility and cardiolipin homeostasis	Mitochondrial dysfunctions	Mitochondrial dysfunctions	Association studies/moderate
RAPSN (Receptor associated protein of the synapse)	rs35705029	Postsynaptic protein, anchors nicotinic acetylcholine receptors	Impairs the cholirogenic neuron functions by acetylcholine deficits	Synaptic dysfunctions in retina	Association studies/moderate
SLC39A13 (Zinc transporter transmembrane protein)	rs755554	Zinc transport among organelles, regulating zinc levels	Impaired zinc homeostasis, zinc accumulation in brain	Zinc accumulation in the retina	Association studies/moderate
CADM2 (Cell adhesion molecule 2)	rs71316816 rs13101042 rs2220243	Involved in formation and maintenance of synapses and cellular adhesion	Synaptic dysfunctions in brain	Impaired cell adhesion in retina and optic nerve impairment	Association studies/moderate

## Data Availability

No new data were created or analyzed in this study. Data sharing is not applicable to this article.

## Abbreviations

**Aβ/Aβ1-40/Aβ1-42**	Amyloid-beta
**AD**	Alzheimer’s disease
**AMD**	Age-related macular degeneration
**APOE**	Apolipoprotein E
**AOO**	Age of onset
**APP**	Amyloid precursor protein
**CAA**	Cerebral amyloid angiopathy
**CSF**	Cerebrospinal fluid
**CST3**	Cystatin C
**CT**	Computed tomography
**EOAD**	Early-onset Alzheimer’s disease
**FDG-PET**	Fluorodeoxyglucose-positron emission tomography
**GRN**	Granulin precursor
**MAPT**	Microtubule-associated protein tau
**MCI**	Mild cognitive impairment
**MMSE**	Mini-Mental State Examination
**MRI**	Magnetic resonance imaging
**PCA**	Posterior cortical atrophy
**PET**	Positron emission tomography
**PiB PET**	Pittsburgh compound B-positron emission tomography
**p-tau**	phospho-tau
**PSEN1**	Presenilin-1
**PSEN2**	Presenilin-2
**RGC-5**	Retinal ganglion cell line 5
**t-tau**	Total tau protein
